# The feasibility and acceptability of implementing video reflexive ethnography (VRE) as an improvement tool in acute maternity services

**DOI:** 10.1186/s12913-022-08713-9

**Published:** 2022-11-03

**Authors:** Siobhan McHugh, Laura Sheard, Jane O’Hara, Rebecca Lawton

**Affiliations:** 1 Present Address: School of Healthcare, University of Leeds, Baines Wing, Leeds, LS2 9JT UK; 2 Present Address: Department of Health Sciences, University of York, York, YO10 5DD UK; 3 Present Address: School of Psychology, University of Leeds, Leeds, LS2 9JT UK

**Keywords:** Healthcare improvement, Video reflexive ethnography, Maternity, Communication, Teamwork, Multi-disciplinary healthcare teams

## Abstract

**Background:**

Video-reflexive ethnography (VRE) has been argued to be an alternative approach to collaborative learning in healthcare teams, more able to capture the complexities of the healthcare environment than simulation. This study aims to explore the feasibility and acceptability of employing VRE as an improvement tool in acute maternity services.

**Method:**

Focused ethnography and semi-structured interviews (*n* = 17) explored the feasibility of employing VRE from the perspective of the researcher-facilitator, and that of the healthcare staff participants. Reflexive thematic analysis was used to generate key themes.

**Results:**

We identified four themes related to feasibility of employing VRE as an improvement approach: *laying the groundwork*; *challenges of capturing in-situ video footage*; *effective facilitation of reflexive feedback*; and, *power to change*. Of note was the central role of the facilitator in building and maintaining staff trust in the process, particularly in being able to guide collaborative, non-punitive discussion during reflexive feedback sessions. Interestingly, when considering implementation of change, structural hierarchies were evident with more senior staff better able to develop and effect ideas.

Two themes related to acceptability of VRE among healthcare staff were identified: *staff response to the role of VRE in improvement*; and *the power of a different perspective*. Staff were overwhelmingly positive about their experience of VRE, particularly appreciating the time, space and autonomy it afforded them to navigate and articulate ideas for change and improvement.

**Conclusion:**

VRE is both feasible and acceptable as an improvement tool with acute, multi-disciplinary maternity staff teams. It is an important healthcare improvement tool that could prompt the development and maintenance of team resilience factors in the face of increasing stress and burn-out of healthcare staff in maternity services.

## Background

Non-technical skills such as communication, decision-making and situational awareness allow healthcare staff to flexibly adapt technical skills to rapidly changing contexts, thus arguably underpin quality and safety of clinical care [1, 2]. Team-based learning of technical and, more recently, non-technical skills has increasingly relied on simulation, often in specially built facilities that attempt to mirror real world contexts as closely as possible [3, 4]. Although successful in prompting collective learning and improvement [5–7], simulation has been criticised for being unable to adequately replicate the complex and challenging physical and social environments in which healthcare teams work [8].

An alternative approach to the development of both technical and non-technical skills is video-reflexive ethnography (VRE); a collaborative visual methodology that supports the need to capture and reflect the local complexities of the healthcare environment [8, 9]. Two of the main principles of VRE are that it is ethnographic, using video footage to capture participants working in situ, and that it is reflexive, prompting collaborative exploration of working practices and/or team-level interactions in context [10, 11]. In its simplest form, VRE involves capturing video footage of healthcare practices and behaviours in situ, editing this footage into short clips that reflect ‘normal’ practice, and playing these clips back to those that embody, apply or experience this practice or behaviour i.e. healthcare staff and potentially patients. As such, VRE embraces the subjectivity and expertise of participants to prompt understanding of specific behaviours and interactions in context [10, 12], and articulation of ideas for locally appropriate practice redesign and improvement [11, 13].

There are strong indications that healthcare-based VRE can support improvement in healthcare by, for example, encouraging practice redesign of clinical handover and ward round [14–16], or eliciting collective learning about and improvement of multi-disciplinary team communication [17, 18]. It has also been used to strengthen infection control practices [19, 20] and to redesign the intensive care unit (ICU) environment [17]. Built on a foundation of collaboration between researchers and participants, VRE offers the potential for prompting learning, change and improvement without a delay in knowledge translation [21].

Despite the espoused success of VRE in prompting improvement within various healthcare settings [11], a recent review of the literature found that there was no explicit assessment of the feasibility or acceptability of implementing VRE with multi-disciplinary healthcare teams in any context [13]. This is most likely to be the result of the positioning of VRE within a post-qualitative research paradigm [11]; thus, the methodological flexibility required to employ VRE creatively and successfully within local contexts, and the importance of participant subjectivity, are not aligned with more traditional evaluation of feasibility and acceptability [22]. Rather than adherence to a strictly defined method to gather objective and generalizable knowledge, VRE enables the formation of dynamic relationships between different actors (researchers, healthcare staff, patients and systems), harnessing their subjective ‘expertise’ to embrace learning and change [22]. More traditional measures of effectiveness, feasibility and acceptability of healthcare improvement approaches - such as reviews, RCTs and survey measures - can lack the level of nuance required to capture such contextual and relational flexibility. Despite this, the uptake of VRE within healthcare is likely to be hampered without ‘evidence’ that it is first feasible and acceptable and, once this is established, that it is an effective approach for improving services [23, 24]. Although some of the potential challenges pertaining to the feasibility and acceptability of employing VRE in healthcare are described in the literature (especially the practicality and acceptance of collecting and storing in situ video data [11]), to date there is no study that directly addresses the question in acute, multi-disciplinary healthcare teams [13]. We address this gap here by considering feasibility and acceptability, harnessing the flexibility of semi-structured interviews and ethnographic field notes to allow us to consider both context and subjectivity and their centrality in the VRE process. Where feasibility concerns how VRE is ‘done’ in context, and acceptability concerns how it is received, understanding the subjective experiences of those involved in such a dynamic and relational approach to improvement is essential in being able to explore feasibility and acceptability.

In this study we explore the feasibility and acceptability of employing VRE as an approach to improving handover practice in an acute multi-disciplinary maternity service. We followed UK MRC guidance on developing and evaluating complex interventions, conducting interviews with staff members and capturing observational data through field notes to answer the following research questions:

1. Is VRE feasible as an improvement approach in acute maternity services?
  - What were the challenges of using VRE with multi-disciplinary healthcare teams in an acute healthcare environment?
  - What were the facilitators to successful use of VRE with multi-disciplinary healthcare teams working in an acute healthcare environment?
2. Is VRE acceptable to multi-disciplinary healthcare teams in acute maternity services?

## Method

We conducted a qualitative study within a maternity delivery suite of a large NHS teaching hospital in the North of England. The study was part of a wider piece of health services research aimed at evaluating VRE as an approach to prompt improvement of teamwork and communication in acute multi-disciplinary healthcare teams (Ethics approval code: *PSYC-170*).

**Fig. 1:**
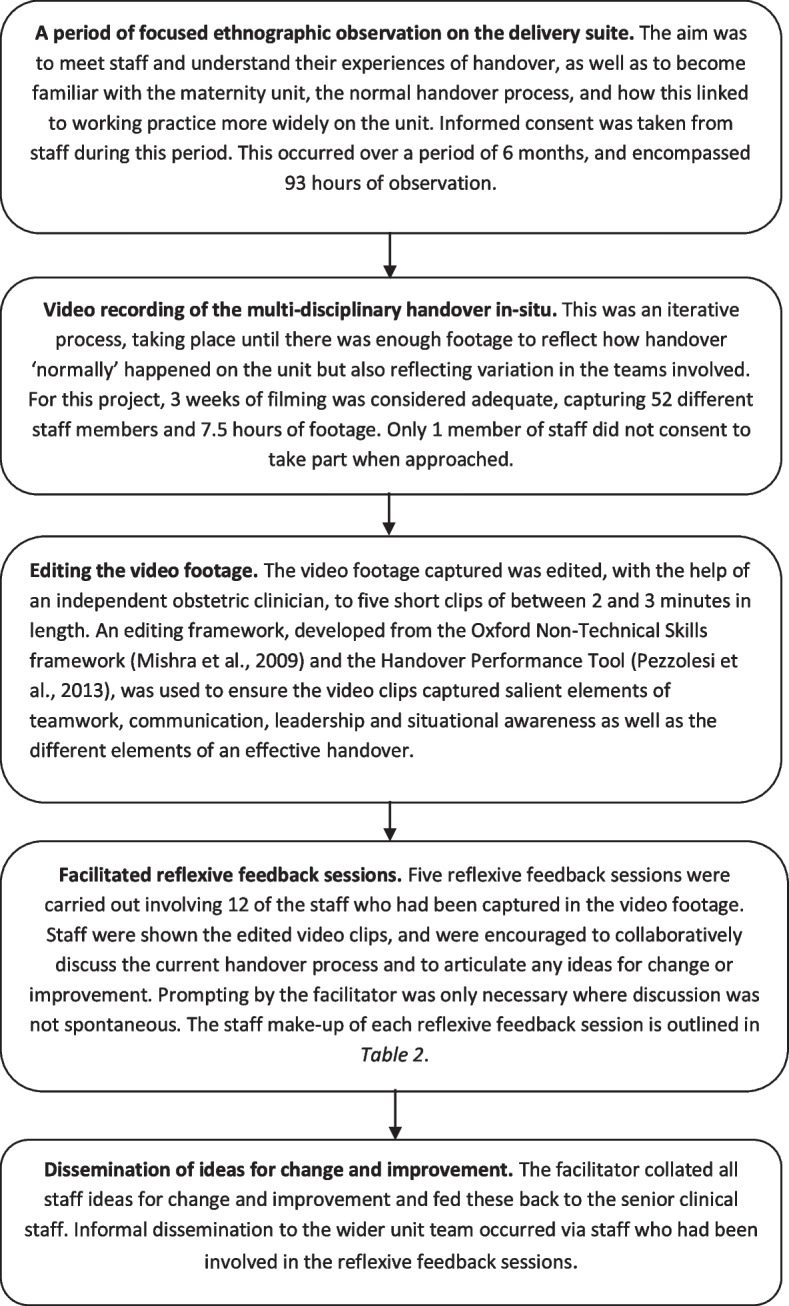
An overview of the VRE process employed in this study as a tool to support improvement of teamwork and communication in multi-disciplinary maternity teams in an acute labour ward

**Fig. 2:**
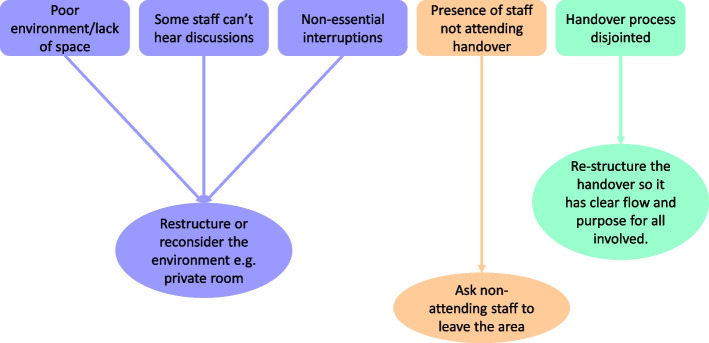
Examples of areas for improvement and solutions for change articulated by staff during reflexive feedback sessions

For context, it is important to consider the dual nature of VRE, spanning the boundary of health services research and quality improvement [25]. In this study, VRE was used as an improvement approach sitting within a wider health services research project that aimed to evaluate its use within acute maternity services. Thus, we had to extricate aspects of feasibility and acceptability specifically related to the application of VRE as an improvement approach from that of conducting a research project in an acute healthcare environment. For example, in the wider research project the process of applying to ethics would have been included in any assessment of feasibility, whereas this is not required for service improvement processes. In its simplest terms, VRE as an improvement approach in this study involved collecting in situ video footage of the multi-disciplinary clinical handover on an acute labour ward, and editing the footage into short 2 to 3 minute clips. The clips were then played back to small groups of healthcare staff who had been involved in the filming in reflexive feedback sessions, where they were encouraged to collectively discuss the current handover process and suggest ideas for change or improvement. A more detailed synopsis of the VRE process as employed in this research is included in Fig. 1. Examples of suggested areas for improvement and ideas for change raised by staff during reflexive feedback sessions are outlined in Fig. 2.

Primary data were drawn from semi-structured interviews and facilitator field notes gathered from 93 hours of ethnographic observation. All participants gave informed consent to take part in this study.

### Semi-structured interviews

Seventeen members of staff took part in semi-structured interviews. The breakdown of staff per job role is included in Table 1. Eleven of the 17 were involved in the reflexive feedback sessions. Table 2 outlines the staff involved in each reflexive feedback session by job role. A further 6 members of staff who were not directly involved in VRE consented to be interviewed about changes to the handover, and the effect of any change on the wider staff team.

**Table 1:** The breakdown of staff per role in the multi-disciplinary team interviewed

Staff Role	Number of staff interviewed
Consultant obstetrician	3
Consultant anaesthetist	2
Midwife coordinator	3
Obstetric registrar	3
Anaesthetic registrar	2
Scrub nurse	3
Operating Department Practitioner (ODP)	1

**Table 2:** The breakdown of staff per role in each reflexive feedback session

Reflexive feedback session	Staff involved
1	Consultant obstetrician Consultant anaesthetist Obstetric registrar
2	Midwife coordinator Obstetric registrar
3	ODP Scrub nurse
4	Midwife coordinator Anaesthetic registrar
5	Consultant anaesthetist Obstetric registrar Scrub nurse

All participants were interviewed following the implementation of changes to the handover, once the VRE process was complete. Participants were asked to retrospectively consider the VRE process, their involvement (if any), and the implementation of any change or improvement. An adaptive topic guide included exploration of:

(i) Individual experience of the VRE process overall;
(ii) Involvement in the VRE process (if any);
(iii) How changes to the MDT handover affected daily working practices;
(iv) Experiences of the way the changes to handover were decided upon and implemented.

In addition to these specific topics included by the researcher, there was also emphasis placed on exploring what was important about the process to participants themselves. Interviews were audio recorded and transcribed verbatim.

### Focused ethnography

A single researcher (SM) conducted periods of focused ethnography on the labour ward before, during and after the VRE process as part of the wider health services research project (see Fig. 1 for more detail). For the purposes of this paper, we have focused on observations related to assessment of feasibility and acceptability. Observations prior to implementation of VRE focused on introducing and setting up the process on an acute labour ward. Those during the process attended to implementation and facilitation of the various elements of the VRE process. Observations after the VRE process focused particularly on how improvements were implemented in context.

### Analysis

The data collected took the form of interview transcripts and a longitudinal researcher field note document collated via expansion of descriptive and conceptual field notes captured in note form on-site. A systematic approach to analysis of the combined data set was taken, where raw data and emergent findings were iteratively visited and revisited to explore concepts related to feasibility and acceptability [26]. Reflexive thematic analysis was guided by a six-phase approach [27, 28]: data familiarisation; initial code generation; constructing themes; reviewing potential themes; defining and naming themes; and reporting results. This approach acknowledged the ideas and concepts brought to the process by the primary researcher (SM) drawn from understanding of the existing literature and their experience as the facilitator. Taking a flexible approach meant that any unanticipated codes could be identified and included. A single researcher (SM) reviewed all field notes and transcripts. A more interpretive, narrative approach [29] to drawing together the results was employed, where the wider research team (RL, LS, JOH) engaged in extensive discussions with the primary researcher (SM) to refine the final themes.

#### Analytic context: feasibility and acceptability defined

Before analysing the combined data set, the concepts of feasibility and acceptability were defined as a primary indicator of their meaning in the context of this research. VRE as a healthcare improvement approach relies on sustained engagement of key stakeholders. Evaluation of feasibility was defined as the most successful practices to give the best chance of success [23]. Important indicators for feasibility were the extent to which VRE could be successfully implemented in acute healthcare environments, the extent to which staff engaged with VRE and the discovery and implementation of improvements. Acceptability was defined as the degree to which the process is satisfactory to those involved [30]. The central challenge raised in the current literature is the acceptability of capturing in-situ working practice on camera, and whether healthcare staff feel the use of video is justified to achieve the aim of the improvement work [11, 31].

## Results

### Feasibility

Four overarching themes related to the feasibility of employing VRE as an improvement approach were identified: *laying the groundwork*; *challenges of capturing in-situ video footage*; *effective facilitation of reflexive feedback*; *power to change*. Considering our definition of feasibility specific to the implementation of VRE in context, the majority of the data contributing to the development of these themes came from researcher field notes.

### Laying the groundwork

#### Importance of clinician engagement

At the beginning of the project our assumption was that the engagement of clinicians in positions of leadership would be critical for staff engagement and confidence. However, while it was important to staff that a clinical lead had agreed for the facilitator to film in-situ, the role of the most senior clinical staff in building confidence about the VRE process among the wider staff team was otherwise minimal. Instead, well-regarded healthcare professionals within the unit who were prepared to champion the VRE process were critical in building wider staff support, and in being able to more specifically articulate any clinically appropriate links between potential improvements and the quality and safety of care.

‘*… [CO] enthusiasm when he’s talking about the project is really infectious with other staff members. I can see straight away how popular he is with his colleagues. Seeing his involvement seems to pique their interest in VRE and how it could lead to improvement. He can sell the project from a staff perspective rather than just coming from a research perspective.’*

[Researcher Field Notes]

*‘I was a bit nervous about saying yes at first because no one wants to see themselves on film do they, especially not in scrubs* [SM: Haha, yeah*] but the fact that [staff names] were involved and how keen they were made me want to do it and be part of improving the way we do things as a team.’*

[Interview, MC2]

Considering VRE as a multi-stage process in which there are periods of intense engagement between the facilitator and staff participants, and periods of time where the facilitator was not always present on site, clinician champions were crucial in maintaining staff engagement during periods where there was less active facilitator presence.

#### Making VRE work in context

It was important for the facilitator to understand and embrace the context of the healthcare environment, and the healthcare practices under scrutiny, in order to successfully plan the practicalities of the VRE process. This was true of all three main stages of VRE; video ethnography, video editing and reflexive feedback sessions. It was only through observing the specific practice – in this case the handover – that the facilitator was able to make practical decisions about the type of camera that would best capture the specific behaviours and interactions in question, and the placement of the camera (which may have implications for who should provide informed consent).

*‘The place where they do the MDT handover is quite difficult to film and it might be difficult to get everyone in the shot. There are two big screens with patient data visible too. I’ve asked the ward manager about attaching the camera to the wall above the screens and I can operate it remotely from my phone.’*

[Researcher Field Notes]

*‘One of the consultant anaesthetists asked straight away where the camera would be placed and what would happen if identifiable patient information was captured.’*

[Researcher Field Notes]

Developing an understanding of the specific healthcare practice of focus also enabled the facilitator to define a clear framework, based on the current literature on MDT handover as well as this contextual knowledge, to guide the video editing process (see Fig. 1). That said, as the facilitator was non-clinical, input from a clinician in addition to the evidence-led framework was invaluable when making the editing decisions to ensure the final clips maintained salient contextual and clinical elements.

*‘[Independent clinician] was really clear during the whole editing process about what was normal practice, and what would be important to keep in the final clips.’*

[Researcher Field Notes]

One of the difficulties in managing a multi-disciplinary approach to the reflexive feedback sessions was the navigation of different shift patterns, roles and responsibilities of specific staff groups, and different levels of flexibility for different staff roles. Maintaining multi-disciplinarity in the reflexive feedback sessions required a level of flexibility in terms of numbers of attending staff and the time of the session.

*‘There were only three staff members in the feedback session today, but the conversation was spontaneous following the footage and flowed well...all staff members seemed to be able to contribute within the feedback session as and when they wished’*

[Researcher Field Notes]

*‘I think, the feedback, I couldn’t make any of the pre-selected times because we don’t get the training time like the doctors do so, erm, I, it was much easier to arrange a time directly with you* **[SM: OK]** *because I could just work it round my shifts…erm…and when it was less likely I’d be caught up.’*

[Interview, MC2]

Maintaining a level of flexibility about the time and staff make-up of reflexive feedback sessions also allowed the facilitator to respond to any concerns or apprehension about watching footage or sharing ideas within a large multi-disciplinary staff group. These particular concerns were more evident when discussing the process with allied health professionals or more junior clinicians. Allowing for smaller reflexive feedback groups meant that these staff felt more confident about speaking up and sharing ideas, even where feedback sessions still maintained multi-disciplinarity across role and level of seniority.

*‘Some of the scrub nurses asked about the size of the feedback sessions today. They were worried about watching themselves and whether they would be able to raise their ideas if there were lots of people. They mentioned that as one of the main problems with the handover. I have reassured them that the groups will be smaller, but that they will still be multi-disciplinary which they seemed happier with.’*

[Researcher Field Notes]

### Challenges of capturing in-situ video footage

#### Gaining trust for staff engagement

The central role of the facilitator in engaging staff was particularly salient. When staff understood the purpose of capturing video footage in-situ, and the process of VRE, they generally expressed interest in being involved. The majority of staff developed an understanding of the VRE process through informal conversations with, and asking questions of, the facilitator. Healthcare staff preferred inter-personal engagement as the primary form of information. Interestingly most staff asked similar questions, particularly linked to the justification for using video footage, and how outcomes might relate to working practice, patient safety and staff well-being.

*‘All of the staff approached in clinic today asked very similar questions about the video footage, including how it would be stored, who would see it, and what the video footage would add to the project that observation or discussion don’t’*

[Researcher Field Notes]

This consistent inter-personal engagement, and mutual appreciation of vulnerability between facilitator and staff, contributed to the development of essential trusting relationships particularly related to filming of in-situ working practices.

*“Some of the midwives and theatre staff asked questions about who would see the footage and how it would be used… They made it clear that they were really worried about it being used for clinical audit or judgement of their individual practice. I felt like just sitting and spending some time chatting to them put them at ease, and by the end of the conversations they seemed really excited that the VRE process would give them a chance to raise, and discuss, their ideas”.*

[Researcher Field Notes]

Interestingly, discussions with staff specifically regarding the use of video allowed for parts of the VRE process to be co-created with them, for example how to capture video footage in-situ, with emphasis on protecting patient information and being sensitive to staff concerns.

#### Consent for filming

Consent to be filmed was an important factor in the initial trust-building between the facilitator and potential staff participants. Consent in this instance was less about a research process, and more a process by which to give staff the autonomy to decide whether or not they wanted to be filmed, an opportunity to ask questions, and set a collaborative tone for the VRE process as a whole.

*‘I’ve had some brilliant discussions with staff today while I was on labour ward taking consent. Initially a lot of staff think it’s just another research project being done to them, but when they realise that they’ll have the opportunity to raise their ideas for improvement that really changes the way they interact with the study information. So many staff today started out nervous about being filmed, but by the end of our discussions they were so excited and positive about being involved. There was a real buzz.’*

[Researcher Field Notes]

The transient nature of staff teams, and the different rotas for obstetric and theatre teams, meant that planning filming based on staff that had provided consent could be undone by last minute shift changes. In an acute healthcare environment, there is little that can be done aside from having flexibility in the filming schedule.

### Effective facilitation of reflexive feedback

The concept of reflexivity is one of the four underpinning principles of VRE [11], thus reflexive feedback is one of the key elements of the VRE process. In these reflexive feedback sessions, selected video clips are shown back to staff, and the facilitator guides collaborative, non-punitive interpretation and discussion between participants about daily working practice and suggested change or improvement. Thus, reflexive sessions are spaces of collaborative knowledge creation, where staff can explicitly engage with and discuss everyday situations, interactions and working practices [9, 11].

#### Setting the tone

Staff participants felt that clarity in the brief instructions given by the facilitator prior to watching the video footage helped them focus on process and structural elements of the handover, rather than discussion of individual behaviours or performance.

*“The way you were so clear in asking us to focus specifically on the process level of the handover, and focus right in on you know the teamwork and communication and how we work together, it meant I didn’t really focus too much on myself which even surprised me”*[Interview, MC1]

Attending to the process and structure of the handover meant that staff felt less apprehensive about contributing to collaborative, group discussion. Setting clear boundaries for discussion also demonstrated awareness of the sensitivities of watching oneself on film, creating a psychologically safe space for staff to navigate and articulate their ideas.

#### Prompting discoveries about work

During the reflexive sessions, the role of the facilitator varied. In some cases staff were quick to identify things they wanted to discuss and discoveries e.g about the way things are done, were unprompted by the facilitator. In other cases, it was necessary for the facilitator to interject, particularly when it came to encouraging discussions about positive aspects of the handover.

*‘I’m really shocked at how little facilitation I had to do in that feedback session (Session 1). Even during the video they were pointing out little things to each other, and as soon as the video finished they just started spontaneously talking together about what they’d seen and how they could improve.’*

[Researcher Field Notes]

*‘As soon as you said at the beginning about looking at processes, I was so focused when I was watching the video then. Erm, I just had so many ideas as I was watching, when it finished it just felt like we were all on the same page straight away pointing things out and all chipping in you know.’*

[Interview, OR2]

Facilitator input was required more frequently in one feedback session, comprised only of nursing and allied health professionals. In feedback sessions that were more mixed, collaborative discussion was observed to flow more readily, although there was no demonstrable difference in the input of more senior staff than those more junior.

*‘I had to prompt a bit more today. After the video finished they all sat in silence, so I just asked a very general question to start them off about whether the video was reflective of what normally happens. This started a discussion about what happens at the moment, but I had to prompt again about ideas for change or improvement. They seemed reluctant to say anything was wrong initially.’*

[Researcher Field Notes]

Staff across all feedback groups were less likely to share positive working practice without direct prompts from the facilitator. This was despite them identifying opportunity to appreciate positive working practice a particular benefit of the VRE process.

*‘It felt a bit unnatural really, you know, like talking about what we do well. We never really get the chance to do that. Everyone focuses on what we aren’t doing, or what we need to do to be better. It felt weird, but really good actually.’*

[Interview, MC1]

### Power to change

Senior staff involvement was important in the implementation of change following the VRE process. Structural hierarchies meant that senior staff were better able to present and sign-off ideas for change or improvement (Fig. 2) at a departmental level. Interaction between senior staff and the wider staff body meant that disparate staff groups were given the time and space to consider and comment on the agreed course of improvement, yet only staff of a certain seniority had the autonomy and power (perceived or actual) to confidently drive and implement these ideas.

*‘The interviews today were difficult at times because it seems that staff below consultant level feel unable to drive any change and so this is an added layer to negotiate when considering how to disseminate the discoveries made by staff, and who we disseminate these results to. It is important to ensure that all staff feel their ideas from this process are valued’*

[Researcher Field Notes]

As such, negotiating autonomy for all staff involved in the reflexive feedback sessions, and more widely any staff that would be affected by planned changes to working practice, was particularly important for the facilitator within the bounds of entrenched perceived hierarchies. Thus, the collation and dissemination of suggested changes discussed across all reflexive feedback sessions to those groups or individuals who have the power to implement change or improvement is an important level of facilitation.

*‘The unit leadership team seemed receptive to the suggested changes to the handover, and they were particularly interested following the successful implementation of the new handover protocol driven by the staff themselves.’*

[Researcher Field Notes]

#### Acceptability

Two overarching themes relating specifically to acceptability of the VRE process were identified from the data; *staff response to the role of VRE in improvement* and *the power of a different perspective*. Considering our definition of acceptability being specific to staff experience of the VRE process, the majority of the data contributing to the development of these themes came from staff interviews.

### Staff response to the role of VRE in improvement

#### Collaboration and shared understanding

Staff were generally positive about VRE. There was a sense of consensus that improvements could be made to the handover, thus the opportunity to view the handover without the pressure of having to attend to clinical information was welcomed. Interestingly, although staff were overwhelmingly positive about the opportunity to identify improvements, they also recognised VRE as an opportunity to view positive elements of working practice.

More generally, staff appreciated the time and space the VRE process afforded them to be able to navigate specific working practices, and articulate their own ideas for change and improvement. They also noted the benefit of collaborative discussion, focusing on the importance of listening to, understanding and exploring different perspectives in furthering their own understanding of the specific structures and processes underpinning everyday working practice.

*‘It was actually really good to feel that someone wanted to listen to our ideas and views on what we do every day rather than telling us what to do or what to change’*

[Interview, SN1]

*‘I couldn’t believe that what I was seeing was the same handover if I’m honest... There were just so many things I could see straight away that I would never have thought about without actually seeing the handover from a different perspective and when I’m not having to think about holding all of this information in my head’*

[Interview, MC2]

Interestingly, positivity about the VRE process was also evident in staff that had not been directly involved in the process itself, but had spoken to colleagues who had. There was a sense that VRE prompted more open conversations within the wider staff body about the handover process and how it might be improved.

*‘I was chatting to one of the regs during lunch and she was so positive about watching the film back and the ideas that had come from her group... It made me wish I’d been on shift at the right time so I could have been involved and seen the handover from that perspective. She’s told me some of the ideas though and I’m thinking more about them all now whenever we handover, where I stand and whether people can hear me and things’*

[Obstetric Registrar, OR1]

#### Autonomy and implementation of improvement ideas

Concerns raised by staff primarily focused on whether and how changes would be implemented following their contributions and ideas in the reflexive feedback sessions. Concerns about structural hierarchies were also raised, especially those between broad disciplines. Reassurance was important that all ideas would be collated and disseminated to groups or individuals that had the power to drive change. This led some staff to question the level of autonomy VRE provided if ideas for change or improvement still had to be ‘signed off’, however they did maintain that creating space for conversations and ideas for change and improvement that were staff-led was an important first step to translating these changes into practice.

*‘It felt good to be able to raise ideas in those group chats with you there* [SM: yeah]*but I suppose, erm, well I, I just didn’t know if our ideas from the theatre team would be given the same weight as the ones from the obstetric team ‘cos it sometimes feels like we need quite different things but this is their domain.*[SM: right, ok]*So yeah, it was good to know everything was fed back but then it was like a question of who would make the decisions after that.’*

[Interview, SN1]

*‘It’s interesting because it feels great to have the space to discuss ideas and plans for improvement, but then what happens to those ideas?*[SM: Ok, yeah]*Are we expected to run with them and try and work out ways of implementing them? I don’t really know so, yeah, it’s a great starting point but now where do we go? I don’t feel like I could just go and start making changes without someone higher up telling me I could.’*

[Interview, OR1]

### The power of a different perspective

It was evident that participants’ perceptions of VRE changed over time as their understanding of the process broadened. Most staff reported feeling more positive about being filmed in situ following participation in the reflexive feedback sessions. Focusing more specifically on the practicalities of VRE, staff also found themselves better able to focus on the more structural elements of the handover due to the viewpoint provided by the fish eye lens of the camera. Making the whole handover environment visible to all staff meant that they were less likely to focus on individual performance and behaviours than they anticipated.

*‘I was so worried about watching back if I looked like I was just stood there not inputting anything I would be embarrassed (SM: ok) but I wasn’t watching myself at all more everyone and the team’*

[Interview, SN2]

*‘I think it helped seeing the handover from above, like, seeing the whole thing it meant I was straight away focusing on the environment and the whole team and how we were communicating together and what that looks like’*

[Interview, MC1]

Interestingly, staff perceptions of the working practice under scrutiny also changed during the process. Enabling staff to view working practice from a different perspective allowed them to shift their focus from a more professionally critical perspective to a more balanced perspective, appreciating the positive elements of working practice as well as identifying wider elements to change or improve. Staff also reported better appreciation of the quality and safety of the care they provided in an environment that was more socially and practically complex than they had been acutely aware of.

*‘At the beginning of the session I remember thinking this is going to be awful because I had quite a negative feeling of handover thinking it was something we have to do...erm...but we don’t really do well, but actually seeing it I was seeing the positive stuff we do as well as things we maybe need to change. I felt more positive coming out of that than going in, and so the next time I was in handover I remember thinking it was ok’*

[Interview, OR1]

*‘After the feedback session I actually felt like I was more positive at work because the video really showed how hard the environment is and actually we do, we really do well to navigate all of that, erm, so yeah I just felt like I was even interacting more positively with people, with other staff and patients because it’s harder than we give ourselves credit for what we do (SM: yeah), and it was nice to think about it positively’*

[Interview, OR2]

## Discussion

The aim of this study was to identify key factors pertaining to the feasibility and acceptability of VRE as a tool for improvement in acute maternity services. A recent review demonstrated a lack of reporting of these factors in the published literature [13]. Four major themes related to feasibility were identified (*laying the groundwork, challenges of capturing video footage in-situ, effective facilitation of reflexive feedback* and *power to change*) and two major themes relating to acceptability (*staff response to the role of VRE in improvement* and *the power of a different perspective)*.

### Factors associated with feasibility

The preliminary stages of implementing VRE are crucial to the success of the process. Leadership buy-in was important and has been identified as key to the success of improvement approaches within the wider healthcare implementation literature [32]. Furthermore, recognising and employing different strategies for presentation and discussion about the use of VRE with different healthcare audiences (e.g. clinicians, managers, patients and families) was key groundwork that allowed for initial trust building. Mutual trust between stakeholders forms important cross-boundary relationships that can engender more rapid social change [14].

Although the current VRE literature makes reference to the challenges of bringing groups of healthcare professionals together where staff are time limited and often over-stretched and thus the importance of a flexible approach [9, 11, 14, 17], the suggestion that organising sessions within already existing structures such as team meetings [11] is at odds with the findings of this study. In fact, our findings primarily suggest the importance of retaining flexibility in timing, location, and size of participant groups when arranging reflexive feedback sessions. It is pertinent to note this may be particularly relevant in acute maternity services or any healthcare context where staff teams are inherently more transient. Furthermore, concerns or apprehension about sharing ideas and collaborative discussion in large multi-disciplinary staff groups must be taken into account and addressed where necessary when arranging reflexive feedback sessions, as real and perceived hierarchies and silos are still inherent in healthcare teams. These more affective dimensions of healthcare improvement approaches, particularly involving multi-disciplinary staff teams, can be overlooked, but are central to the success of VRE.

In line with previous literature, care for participants is imperative to the success of the reflexive feedback sessions [9, 11]. In particular, watching oneself on film and the discussion of potential issues could be construed by staff as personal risk taking within their organisational teams. Staff recognised the importance of the facilitator in providing and maintaining a safe space throughout the feedback session, directly guiding staff to place particular emphasis on structural and process factors rather than individual behaviours when watching the footage. This is important when considering team learning behaviours, and the positive association between team psychological safety and collective learning [33, 34].

Lack of clarity about how ideas from the reflexive feedback sessions would be disseminated and implemented was linked to the notion of structural power and the proliferation of steep hierarchies still present within healthcare teams [35]. These hierarchical gradients were particularly apparent between obstetric and theatre staff, although many junior obstetric staff did not feel they were in a position to affect change. However, hierarchies were more practically than socially driven. Staff might feel more able to articulate their ideas to a more senior colleague, but they rarely feel in a position to drive change themselves [36]. Participatory methods, such as VRE, can prompt a more dynamic representation of power to participants which elicits both positive and negative affect. It is therefore important to consider, that even in teams with the most positive culture, there may be organisational structures that cause the perpetuation of hierarchies and silos [37].

### Factors associated with acceptability

It was evident that healthcare staff participants were acutely aware of the different perspective offered by the use of in-situ video footage. In line with the current literature suggesting that VRE has a ‘hologramatic’ effect, staff were able to see past their own individual performance to appreciate the complexity and intricacy of the handover process within a multi-disciplinary team [9, 11]. This led to a renewed appreciation of the VRE process following the reflexive feedback session and an increasingly positive understanding of what the process could offer. Staff perspectives on the handover process also became more positive throughout the process. The opportunity to consider the positive elements of their work, as well as ideas for change or improvement, was an important outcome of the VRE process among maternity staff participants, regardless of any tangible outcomes. This is particularly pertinent in the current climate, where UK maternity services have been the focus of a number of high-profile independent inquiries in recent years [38, 39]. More generally, as healthcare systems globally face significant and mounting challenges, there is consistent and growing evidence that the burden is disproportionately falling on clinical front-line healthcare professionals. Increasing complexity of care, combined with the proliferating effects of the COVID-19 pandemic, have resulted in high levels of stress and burnout among staff [40]. The negative psychological effects of the increasing burden on clinical healthcare staff has been linked consistently to healthcare quality and patient safety outcomes [40]. Thus providing staff on the clinical front-line with the opportunity to view themselves working safely within (and often despite) the complexity of the local care environment, and the autonomy to identify ideas for change and improvement, could arguably support individuals and teams to continually adapt, learn and improve whilst also developing capacity to respond to individual and collective contextual stressors [40, 41].

The main concern with regard to the acceptability of VRE from a staff perspective was the translation of discoveries and ideas created within a space for collective discussion into tangible improvement. Iedema et al. [11] suggest in their guidelines that perspectives on what should be improved might be divergent and, as such, evaluation of outcomes should be considered within the context of the local factors that might have shaped such outcomes. That said, there is little guidance within the current literature that explores how researchers can navigate the interface between the solutions discussed by all staff in the reflexive feedback sessions and the implementation of appropriate solutions for improvement. It is important that future research attempts to understand how best to negotiate change and improvement in an equitable way for all staff.

### Limitations

Limitations of this study include that the researcher interviewing the participants was also the facilitator of the VRE process, so any concerns about the process, particularly facilitation of the reflexive feedback sessions, might not have been raised by staff. Although this study included a small sample size for the reflexive feedback sessions relative to the number of staff contracted to work on the labour ward, staff working in acute healthcare environments are already time-poor, and there was good representation across participant groups.

### Summary

The findings of this study provide a novel lens on the feasibility of VRE as an improvement tool in acute maternity services. VRE is a positive experience for staff, particularly the time and space afforded to consider their working practices, and the autonomy to suggest or prompt locally-appropriate change or improvement. Where healthcare improvement often requires staff to do additional work, this study also suggests that VRE could provide a low-cost, low-demand process, harnessing understanding of ‘work-as-done’ in context by which to develop both individual and team resilience factors. This low burden of involvement is particularly important in the face of increasing burn out and stress in front-line clinical healthcare staff. The study outlines key considerations when using VRE as an improvement method with acute multi-disciplinary healthcare teams, particularly the importance of building and maintaining relationships, and the nuances of the practicalities of employing VRE. Future research should focus on exploration of how to support equitable power to change, particularly in the face of organisational or systemic factors that might support or prevent dissemination and implementation of improvement ideas. It is also important to explore in more detail the mechanisms by which in-situ video footage and effective facilitation can prompt the discovery and articulation of specific ideas for change and improvement. In addition, this study suggests the importance of flexibility in how we approach the evaluation of feasibility and acceptability of healthcare improvement methods. As a dynamic and relational system, our understanding of improvement approaches must take into account different healthcare contexts (both human and environmental), and how this can affect how we ‘do’ improvement, and how such approaches can affect those involved.

## Data Availability

The datasets used and/or analysed during the current study are available from the corresponding author on reasonable request.
