# Correction: The feasibility and acceptability of implementing video reflexive ethnography (VRE) as an improvement tool in acute maternity services

**DOI:** 10.1186/s12913-023-09234-9

**Published:** 2023-03-14

**Authors:** Siobhan McHugh, Laura Sheard, Jane O’Hara, Rebecca Lawton

**Affiliations:** 1 Present Address: School of Healthcare, University of Leeds, Baines Wing, Leeds, LS2 9JT UK; 2 Present Address: Department of Health Sciences, University of York, York, YO10 5DD UK; 3 Present Address: School of Psychology, University of Leeds, Leeds, LS2 9JT UK

**Correction to: BMC Health Services Research (2022) 22:1308.**

10.1186/s12913-022-08713-9.

Following publication of the original article [1], the authors reported missing information in the ‘Acknowledgements’ section.

The statement in the ‘Acknowledgements’ section originally read: None.

The statement in the ‘Acknowledgements’ section should read: The authors would like to thank Mr Dileep Wijeratne for his extensive support in both the set up and delivery of this research project. We would also like to thank all of the staff from Leeds Teaching Hospitals maternity services, in particular the clinical leads for anaesthetics, obstetrics and the head of midwifery for their time and support.

The original article [1] has been updated.
